# Seasonal trends in lysogeny in an Appalachian oak-hickory forest soil

**DOI:** 10.1128/aem.01408-23

**Published:** 2023-12-12

**Authors:** Melaina L. Jacoby, Graham D. Hogg, Madeleine R. Assaad, Kurt E. Williamson

**Affiliations:** 1 Center for Vaccine Research, University of Pittsburgh, Pittsburgh, Pennsylvania, USA; 2 American Type Culture Collection, Manassas, Virginia, USA; 3 Biology Department, College of William & Mary, Williamsburg, Virginia, USA; Unversidad de los Andes, Bogotá, Colombia

**Keywords:** phage, soil, lysogeny, prophage, induction, mitomycin C, bacteria

## Abstract

Since 1989, investigations into viral ecology have revealed how bacteriophages can influence microbial dynamics within ecosystems at global scales. Most of the information we know about temperate phages, which can integrate themselves into the host genome and remain dormant via a process called lysogeny, has come from research in aquatic ecosystems. Soil environments remain under-studied, and more research is necessary to fully understand the range of impacts phage infections have on the soil bacteria they infect. The aims of this study were to compare the efficacy of different prophage-inducing agents and to elucidate potential temporal trends in lysogeny within a soil bacterial community. In addition to mitomycin C and acyl-homoserine lactones, our results indicated that halosulfuron methyl herbicides may also be potent inducing agents. In optimizing chemical induction assays, we determined that taking steps to reduce background virus particles and starve cells was critical in obtaining consistent results. A clear seasonal trend in inducible lysogeny was observed in an Appalachian oak-hickory forest soil. The average monthly air temperature was negatively correlated with inducible fraction and burst size, supporting the idea that lysogeny provides a mechanism for phage persistence when temperatures are low and host metabolism is slower. Furthermore, the inducible fraction was negatively correlated with both soil bacterial and soil viral abundance, supporting the idea that lysogeny provides a mechanism for temperate phage persistence when host density is lower. The present study is the first of its kind to reveal clear seasonal trends in inducible lysogeny in any soil.

**IMPORTANCE:**

Lysogeny is a relationship in which certain viruses that infect bacteria (phages) may exist within their bacterial host cell as a segment of nucleic acid. In this state, the phage genome is protected from environmental damage and retains the potential to generate progeny particles in the future. It is thought that lysogeny provides a mechanism for long-term persistence for phages when host density is low or hosts are starved—two conditions likely to be found in soils. In the present study, we provide the first known evidence for a seasonal trend in lysogeny in a forest soil. Based on clear relationships observed between lysogeny, temperature, and soil microbial abundance, we find support for previous hypotheses regarding the factors governing lysogeny.

## INTRODUCTION

Viruses are incredibly abundant in Earth’s biosphere. Current estimates place global viral abundance at 10^31^ particles, 12 times the estimated total number of prokaryotes (1). In fact, there are more viruses in a liter of coastal seawater than there are people on the planet (2). Viruses are highly diverse, partially owing to rapid evolution in response to changing selective pressures. As a result, viruses can be found in just about every ecological niche on Earth (3). Bacteriophages, or viruses that infect bacteria, are the dominant virus type in most environmental samples and play important roles in regulating microbial community composition (4), carbon and nutrient cycling, biogeochemical cycles (4, 5), and even the shaping of global climate (5). Studies of viral impacts on these phenomena have been mainly conducted in aquatic ecosystems, where bacteriophage abundance is known to be high, up to 10^8^ mL^−1^ (6), and on average, 10 times higher than bacterial abundance (7). However, an estimated 97% of global viral abundance is located in soils and sediments (1), and more research is necessary to determine whether the models of viral impacts developed predominantly in marine systems can be applied to soils.

Bacteriophages in marine systems may exhibit different replication strategies compared to those in soils due to differences in spatial structure and contact rate between phages and their hosts in these two systems. After infecting a host, a phage can follow one of several replication pathways, depending on its genetic capabilities. The lytic pathway involves hijacking the host’s replication machinery to immediately begin the process of copying phage genomes, expressing phage genes, and assembly of mature phage particles (8). This eventually leads to the lysis of the host cell and the release of progeny phage particles. Lytic replication is believed to provide an evolutionary advantage in marine ecosystems where host abundance and productivity are high, as there are plenty of resources for the phage to replicate successfully and maximize the production of progeny (9). On the other hand, soil ecosystems may offer more limited resources and opportunities for phage replication, severely constraining the number of progeny that can be generated via the lytic cycle. Temperate phages may parlay these disadvantages by replicating via the lysogenic pathway. In lysogeny, a phage-encoded repressor blocks the expression of phage genes involved in lytic replication. The phage genome may also be incorporated into the host’s chromosomal DNA via a phage-encoded integrase (8). In this lysogenic state, the phage genome is stably maintained within the host cell and is termed a prophage.

Lysogeny can last many generations of cell division, producing daughter cells that are all lysogens, themselves. While stable, however, lysogeny is not permanent and can be terminated through a process known as induction, in which extracellular signals trigger a change in prophage (and host) gene expression. One well-characterized signal that can terminate lysogeny is DNA damage to the host cell. Through cellular DNA damage, whether from UV light exposure, chemical mutagens, or toxic compounds, the bacterial SOS response is activated, leading to the degradation of the prophage repressor and transcriptional activation of phage genes required for lytic replication (8). Of course, not all infections by temperate phages will result in lysogeny. For temperate phages, progression through the replication pathway is influenced by environmental and host signals, including host metabolic activity signaled by proteases and nutrient availability, host density signaled through concentrations of quorum-sensing compounds, and host gene expression (7, 8, 10–12). The prevailing view is that temperate phages enter into lysogeny when hosts are starved or host density is low and essentially “wait” until chances of maximizing reproduction have improved (10, 12, 13).

Given that temperate phages have complex genetic circuits that allow them to sense environmental conditions, it follows that variation in factors, such as nutrient availability, weather conditions, and microbial community composition, may ultimately determine the replication pathway of temperate phages, enabling them to maximize either survival or production of progeny. Chemical induction assays are the main way in which the importance of lysogeny as a replication strategy has been determined (14). In aquatic environments, attempts at determining the prevalence of lysogeny through induction assays have been met with variable results. A study of ice-covered Antarctic lakes reported very high levels of lysogeny, with up to 89.5% of the bacterial community containing inducible lysogens (15). In contrast, one study of coastal seawaters found no evidence of inducible lysogeny in any sample (16). Reports from some marine systems suggest a seasonal pattern in lysogeny. Studies conducted in Tampa Bay, FL, USA, indicated an increased proportion of lysogens in the colder, winter months (17, 18), and a study of saline Antarctic lakes reported the highest incidence of lysogeny in winter and spring and a decline in summer (19). While dozens of studies have attempted to characterize the factors governing lysogeny in aquatic systems (14), much less is known regarding lysogeny in soils.

Soil environments are chemically, physically, and biologically diverse at a global scale, and nutrient availability seems to be strongly correlated with both geographic location and local plant characteristics (20). It has also been shown that increased nitrogen and phosphorus input can change the composition of soil microbial communities (21, 22) and that soil depth plays a role in bacterial community composition (23). Based on the heterogeneity of soil environments around the globe, it would follow that such diverse environments would select for viral communities and replication strategies best suited to those environments.

Early theoretical analyses of soil bacteriophage ecology proposed that the lysogenic replication pathway should offer an evolutionary benefit to phages in soil environments, as typical soil characteristics such as long periods of host inactivity and limited opportunities for movement would render exclusively lytic replication suboptimal (10, 24). This hypothesis has gained support over the years as more studies on lysogeny in soils have been conducted. Estimates of inducible fractions of bacteria have ranged from 30% in Delaware soils (25) to 4.6%—21.1% in Antarctic soils (26). A study conducted using five different soils from the Kellogg Biological Station’s (KBS) Long Term Ecological Research site reported inducible fractions of soil bacteria ranging from 1% to 77% (27), and one recent study suggests that the fraction of lysogenic bacteria increases with soil depth (28). In spite of these intriguing results suggesting that lysogeny in soil bacteria is common, there are still very few studies that have focused on lysogeny in soils, resulting in a lack of information on the factors governing this phenomenon.

In most studies to date, mitomycin C has been the gold standard of inducing agents. Mitomycin C is an alkylating agent that cross-links complementary strands of DNA and thus inhibits DNA synthesis (29). Earlier studies, particularly with the *Escherichia coli* (λ) system, indicated that ultraviolet light (UV) can be used as an inducing agent (30, 31), but more recent studies suggest that UV is less effective of an inducing agent than mitomycin C (32). Beyond mitomycin C, there is also interest in identifying environmentally relevant compounds or signals that act to stimulate prophage induction (33–36). Previous, unpublished work from our lab investigated the prophage-inducing potential of the halosulfuron methyl herbicide, SedgeHammer (Gowan Company, Yuma, AZ, USA), which is used on the campus of William and Mary to eliminate nutsedge, a nuisance weed (37). SedgeHammer inhibits the acetolactate synthase enzyme, blocking the production of three amino acids required for DNA replication. In this preliminary work, SedgeHammer treatment of freshwater bacteria stimulated significant increases in viral direct counts, suggesting its potential use as an inducing agent for environmental samples. Finally, bacterial quorum-sensing molecules called acyl-homoserine lactones (AHLs) have been successfully used to induce both *E. coli* (λ) lysogens and bacterial communities from soils (13). The fact that quorum-sensing compounds such as AHLs can induce prophages supports the hypothesis that host density plays a role in whether a phage replicates through the lytic or lysogenic cycle, as quorum sensing itself is a cell-density-dependent phenomenon. Efforts to identify environmentally relevant inducing agents will be helpful to optimize future work studying lysogeny, particularly in soils.

The main goals of the present work were to (i) compare the efficacy of different inducing agents in populations of bacterial lysogens, including *E. coli* (λ) lysogens, natural populations of freshwater bacteria, and natural populations of soil bacteria; (ii) test for seasonal trends in inducible lysogeny in the bacterial community of an Appalachian oak-hickory forest soil; and (iii) determine the factors associated with inducible lysogeny in soil. The results of this work will provide insight into the influence of lysogeny in understudied soil environments, as well as recommendations for improving the reproducibility of induction assays using environmental bacterial populations in future studies.

## MATERIALS AND METHODS

### Sources of bacteria

*Escherichia. coli* W3104 was purchased from Carolina Biological Supply Co. (Burlington, NC, USA). This strain of *E. coli* is a lambda lysogen and should produce phage particles upon induction. *E. coli* W3104 was grown in tryptic soy broth (TSB, Fisher) at 37°C, 150–200 rpm. Soil bacteria were extracted from a loamy sand soil (Emporia Complex) under Appalachian oak-hickory forest in Williamsburg, VA, USA (38). Freshwater bacteria were obtained from Lake Matoaka in Williamsburg, VA, USA (39). Details on sample collection and processing follow below.

### Sample collection

For each soil sample, approximately 200 g of soil was collected with a hand trowel that had been disinfected with 70% ethanol. A composite sample was generated via a random walk method covering a 10 m^2^ plot and placed into a quart-sized Ziploc plastic bag. Soil was then transported to the lab and immediately sieved to 4 mm before extracting bacteria or virus particles. For freshwater samples, 2 L of surface water was collected by hand in a clean polycarbonate bottle that had been triple-rinsed with surface water prior to filling. Water was transported to the lab for immediate use in induction experiments.

### Soil bacterial extraction

Extraction of soil bacteria was carried out in triplicate, as described in reference (40). Briefly, 10 g of sieved soil was added to pre-chilled blender cups on ice, and 100 mL of chilled (4°C) 0.01% sodium deoxycholate (Fisher) was added to each blender cup. Samples were blended on high for 3 minutes and the resulting slurry was allowed to settle on ice for approximately 1 minute. Fifteen milliliters of the resulting slurry was then carefully layered over 5 mL of Nycodenz (1.3 g mL^−1^) in sterile 35 mL polypropylene Oak Ridge tubes. Tubes were then centrifuged at 8,000 × *g* at 4°C for 20 minutes to sediment soil particles. The resulting supernatant was decanted from each tube, pooled in two sterile 50 mL centrifuge tubes, and homogenized by pouring back and forth between the two tubes. Aliquots of soil bacterial extracts were either frozen for bacterial enumeration (transferred to cryovials before snap-freezing in liquid nitrogen and storage at −80°C; nominal storage time, 6 months) or immediately used in prophage induction experiments.

Soil chemical and physical analyses were completed by Waypoint Analytical (Richmond, VA, USA). Soil pH was determined using the 1:1 method in water; soil nitrate, phosphate, calcium, and potassium were determined using ICP-OES of Mehlich-3 soil extracts, and soil organic matter was determined by loss on ignition (Table 1).

**TABLE 1:** Soil properties and related metadata

Date	OM^*a*^ (%)	K (mg kg^−1^)	Mg (mg kg^−1^)	Ca (mg kg^−1^)	pH	CEC^*b*^	NO_3_ (mg kg^−1^)	%W^*c*^ (SD)	Rain^*d*^ (mm)	Air temp^*e*^, °C (SD)
Sept 2021	6.2	40	70	397	5	4.5	1	11.54 (1.367)	65.86	22.1 (3.73)
Oct 2021	6.4	39	82	512	5.3	4.8	1	33.21 (1.024)	142.51	18.4 (3.91)
Nov 2021	6.6	41	96	722	5.2	6.8	1	10.71 (0.171)	17.12	9.36 (4.97)
Dec 2021	7.3	39	73	416	4.7	5.6	1	18.27 (0.685)	34.3	9.39 (5.48)
Jan 2022	5.2	39	64	364	5.4	3.5	2	33.63 (0.561)	122.46	2.93 (5.85)
Feb 2022	7.3	54	98	637	5.3	6	1	31.33 (0.661)	31.7	6.94 (5.05)
Mar 2022	6.9	53	96	631	5.1	6.6	1	32.16 (0.783)	119.66	11.51 (6.4)
Apr 2022	5.2	54	87	520	5.3	5.1	6	27.79 (0.215)	65.44	14.9 (5.51)
May 2022	6	53	82	477	5.8	4	1	24.39 (0.467)	95.68	19.79 (4.99)
June 2022	6.3	44	82	462	5.2	4.7	1	14.83 (0.167)	76.35	23.73 (3.82)
July 2022	6.4	39	82	502	5.4	4.6	3	17.51 (0.652)	154.79	26.34 (3.02)
Aug 2022	6.3	40	84	459	5	5.2	1	17.49 (0.863)	52.4	25.01 (3.37)
CV^*f*^	10.6	15.1	12.6	20.9	5.17	19.4	89.8	38.1	55.6	49.1

^*a*^ Organic matter content, by loss on ignition.

^*b*^ Cation exchange equivalents, in meq 100 g^−1^.

^*c*^ Gravimetric water content.

^*d*^ Average monthly rainfall.

^*e*^ Average monthly air temperature.

^*f*^ Coefficient of variation (%) over the annual observation period.

### Soil viral extraction

Extraction of soil viruses was carried out in triplicate, as described in reference (40). Briefly, 5 g of sieved soil was weighed into sterile 35 mL polypropylene Oak Ridge tubes, and 15 mL of chilled (4°C) 1% potassium citrate buffer (per liter: 10 g potassium citrate, 1.44 g Na_2_HPO_4_·7H_2_O, 0.24 g KH_2_PO_4_, pH 7) was added to each tube. Samples were sonicated for 3 minutes on ice, with each minute interspersed with 30 s manual shaking. Tubes were then centrifuged at 8,000 × *g* at 4°C for 20 minutes to sediment soil particles. The resulting supernatant was filtered through 0.22 µm polyether sulfone (PES) syringe filters, and the filtrate was collected in sterile cryovials. Soil viral extracts were snap-frozen in liquid nitrogen and stored at −80°C until slides were prepared (nominal storage time, 6 months).

### Inducing agents

Mitomycin C was purchased from two different vendors: Thermo Fisher Scientific (Waltham, MA, USA) and ApexBio (Houston, TX, USA); irrespective of source, mitomycin C was added to induced samples at a final concentration of 0.5 µg mL^−1^. SedgeHammer (Gowan Company, Yuma, AZ, USA) herbicide was prepared by dissolving 0.5 g of SedgeHammer in 10 mL of sterile deionized water. SedgeHammer was added to induced samples at a final concentration of 0.05 µg mL^−1^ (37). An equimolar mixture of acyl-homoserine lactones (*N-*Hexanoyl-L-homoserine lactone and *N-*Tetradecanoyl-DL-homoserine lactone, Sigma-Aldrich, St. Louis, MO, USA) was used, and the AHL mixture was added to induced samples to achieve a final concentration of 1 µM ((13).

### Comparison of inducing agents

For comparisons of inducing agents, 30 sterile 50 mL tubes were prepared; 15 tubes received 12 mL of fresh soil bacterial extraction (collected on 1 March 2020), and 15 tubes received 12 mL of whole water from Lake Matoaka (collected on 1 March 2020); soil and water samples were collected as previously described. All induction treatments were set up in triplicate for both soil extracts and freshwater samples: Fisher mitomycin C, ApexBio mitomycin C, SedgeHammer, and the AHL mixture. The remaining three tubes for both soil extracts and freshwater samples were designated as controls and received an equivalent volume (12 µL) of sterile water rather than an inducing agent.

An additional set of experiments was performed with *E. coli* W3104. An aliquot (1 mL) of an overnight culture of *E. coli* W3104 was inoculated into a fresh TSB medium and incubated with shaking for 4 hours. Initial induction experiments using the exponential phase cultures failed due to high levels of virus particles in controls; no statistically significant difference was observed between controls and any of the inducing agents. To reduce background viral abundance from autoinduction, the following steps were implemented. After growing cultures to the early exponential phase, cells were pelleted by centrifugation at 3,000 × *g* for 5 minutes at 20°C. The supernatant was removed by aspirating with a sterile serological pipette and the cells were resuspended in 1× M9 salts (41). The resuspended cells were divided into nine 10 mL portions, each in a sterile 50 mL tube. All inductions were set up in triplicate: ApexBio mitomycin C was added to a final concentration of 0.5 µg mL^−1^, and SedgeHammer was added to a final concentration of 0.05 µg mL^−1^. The remaining three tubes were designated as controls and received an equivalent volume (5 µL) of sterile water rather than an inducing agent.

All samples were blinded (coded) to prevent the identification of treatments/controls. All samples were wrapped in foil to prevent light exposure and placed in a rotary shaker at 140 rpm, 25°C for 24 hours. Following this incubation period, 1 mL aliquots of each sample were transferred to sterile cryovials, frozen in liquid nitrogen, and stored at −80°C until slide preparation (nominally, 1–4 weeks).

### Investigation of temporal trends in soil prophage induction

From September 2021–August 2022, soil samples were collected monthly (between the 1st and 15th of each month) as previously described. Bacteria and virus particles were extracted as previously described. The homogenized bacterial extracts were centrifuged at 3,000 × *g* for 5 minutes at room temperature to pellet cells. The supernatant was aspirated using a sterile serological pipette and the cells were resuspended in 1× M9 salts. The resuspended cells were divided into six 10 mL portions, each in a sterile 50 mL tube. Three tubes received ApexBio mitomycin C (final concentration of 0.5 μg mL^−1^) and three tubes received sterile water (controls). All tubes were wrapped in foil to prevent light exposure and placed in a rotary shaker at 140 rpm, 25°C for 24 hours. Following this incubation period, 1 mL aliquots of each sample were transferred to sterile cryovials, frozen in liquid nitrogen, and stored at −80°C until slide preparation (nominally, 1–4 weeks).

### Slide preparation

Frozen samples were thawed in a warm water bath (~50°C) and then stored on ice. For comparison of inducing agents and investigation of temporal trends in prophage induction from soil bacteria, 20 µL of each sample was suspended in 80 µL sterile deionized water to make up 100 µL total. For the enumeration of ambient soil viral abundance from soil viral extracts, 5 µL of viral extract was suspended in 95 µL of sterile deionized water. Suspended samples were immobilized on Whatman Anodisc filter membranes (13 mm diameter, 0.02 µm pore size, Whatman, Maidstone, UK) held in 13 mm polypropylene Swinnex filter holders. Anodisc filters were prepared and mounted on slides as described in reference (40) with the following amendments: (i) after the sample had been drawn through the filter, one volume (100 µL) of sterile deionized water was added and also drawn through the filter; (ii) after the SYBR Gold solution was drawn through the filter, one volume (100 µL) of sterile deionized water was added and also drawn through the filter.

For the enumeration of ambient soil bacterial abundance from soil bacterial extracts, 25–30 μL of bacterial extract was suspended in sterile deionized water to make up 1 mL total volume. Suspended cells were applied to 25 mm diameter, 0.22 µm pore size black polycarbonate filters (Pall) as described in reference (40). All slides were either analyzed immediately or stored at −20°C until analysis (nominally, 1–3 days).

### Epifluorescence microscopy

Slides were viewed using an Olympus BX51 microscope (Olympus, Center Valley, PA, USA) fitted with an Olympus U-RFL-T mercury lamp, FITC excitation filter, and 100×/1.30 oil lens. Fifteen fields per replicate were digitally photographed at ×1,000 magnification with a Hamamatsu C8484 CCD camera. Efforts were made to select fields of view randomly as to properly sample the potential variation within each slide. Photos were captured and analyzed using either MetaMorph software (MetaMorph, Nashville, TN, USA) or ImageJ MicroManager 2.0 (42).

### Calculations

1. Prophage induced (*P*_*i*_) = VDC_induced_ – VDC_control_, in which VDC is the average viral direct count in the induced or control samples.
2. Bacteria lysed (*B*_*l*_) = BCD_control_ – BDC_induced_, in which BDC is the average bacterial direct count in the induced or control samples.
3. Burst size (*B*_*z*_) = *P*_*i*_/*B*_*l*_, in which *P*_*i*_ is the number of prophages induced and *B*_*l*_ is the number of bacterial cells lysed.
4. Inducible fraction (IF) = (*P*_*i*_/*B*_*z*_) / BDC_control_ × 100, in which *P*_*i*_ is the number of prophages induced, *B*_*z*_ is the calculated burst size, and BDC_control_ is the average number of bacterial cells in the control sample.

Inducible fraction is the percentage of cells in the sample, which are capable of being chemically induced. Previous studies have used the terms “lysogenic fraction” and “fraction of chemically inducible cells” (14) to describe this phenomenon, but we will use the term “inducible fraction” since this is a more accurate description of the data being collected during this study.

### Statistical analyses

All statistical analyses were conducted using GraphPad Prism software (GraphPad, San Diego, CA, USA). For comparisons of inducing agents, one-way ANOVA with Tukey’s multiple comparison *post hoc* test was used to test for significant differences across treatments. For monthly samples, two-tailed unpaired *t*-tests were conducted to compare controls to induced treatments. Significance was defined as *P* **≤** 0.05. Spearman correlation was used to test for relationships between soil properties, including biological measurements [e.g., prophage induced, bacterial abundance, viral abundance, and inducible fraction (Table 2)] and physicochemical measurements (e.g., gravimetric water content, organic matter content, and soil nutrients).

**TABLE 2:** Induction of extracted soil bacteria, associated burst sizes, and inducible fraction^*e*^

Date	Prophage induced 10^5^ (SD)	Percent change (%)^*a*^	*P*-value^*b*^	*B*_*z*_ (error)	IF_Bz_ (error)^*c*^	IF_20_ (error)^*d*^
Sept 2021	18.6 (3.27)	800	0.0060	0.73 (0.55)	49.85 (5.16)	1.81 (0.086)
Oct 2021	119 (85.7)	2,000	0.0016	5.59 (5.66)	56.81 (10.0)	15.93 (1.37)
Nov 2021	107 (22.7)	1,800	0.0021	8.33 (2.83)	50.91 (2.04)	15.28 (0.30)
Dec 2021	116 (11.3)	462	<0.0001	3.89 (1.24)	73.34 (3.86)	14.26 (0.39)
Jan 2022	108 (20.1)	446	0.0007	3.61 (0.91)	75.57 (3.53)	15.11 (0.34)
Feb 2022	98.1 (12.3)	337	0.0002	4.25 (0.68)	67.81 (1.55)	14.92 (0.17)
Mar 2022	106 (24.3)	229	0.0014	4.37 (1.65)	69.27 (3.53)	15.12 (0.39)
Apr 2022	52.6 (23.2)	376	0.0172	3.81 (1.73)	54.48 (3.45)	8.09 (0.30)
May 2022	64.2 (8.57)	79	0.0100	4.39 (2.83)	43.38 (3.61)	9.53 (0.34)
June 2022	11.3 (19.4)	28	0.3691	ND	ND	ND
July 2022	9.99 (5.65)	11	0.7488	ND	ND	ND
Aug 2022	0.598 (2.41)	7	0.4172	ND	ND	ND

^*a*^ Calculated as the percentage increase relative to the average VDC of the corresponding controls.

^*b*^ *P*-value associated with the difference between average VDC in induced samples compared to corresponding controls.

^*c*^ Inducible fraction as a function of the calculated burst size.

^*d*^ Inducible fraction using an assumed burst size of 20.

^*e*^ ND, not determined.

## RESULTS

### Comparison of inducing agents

**Fig 1:**
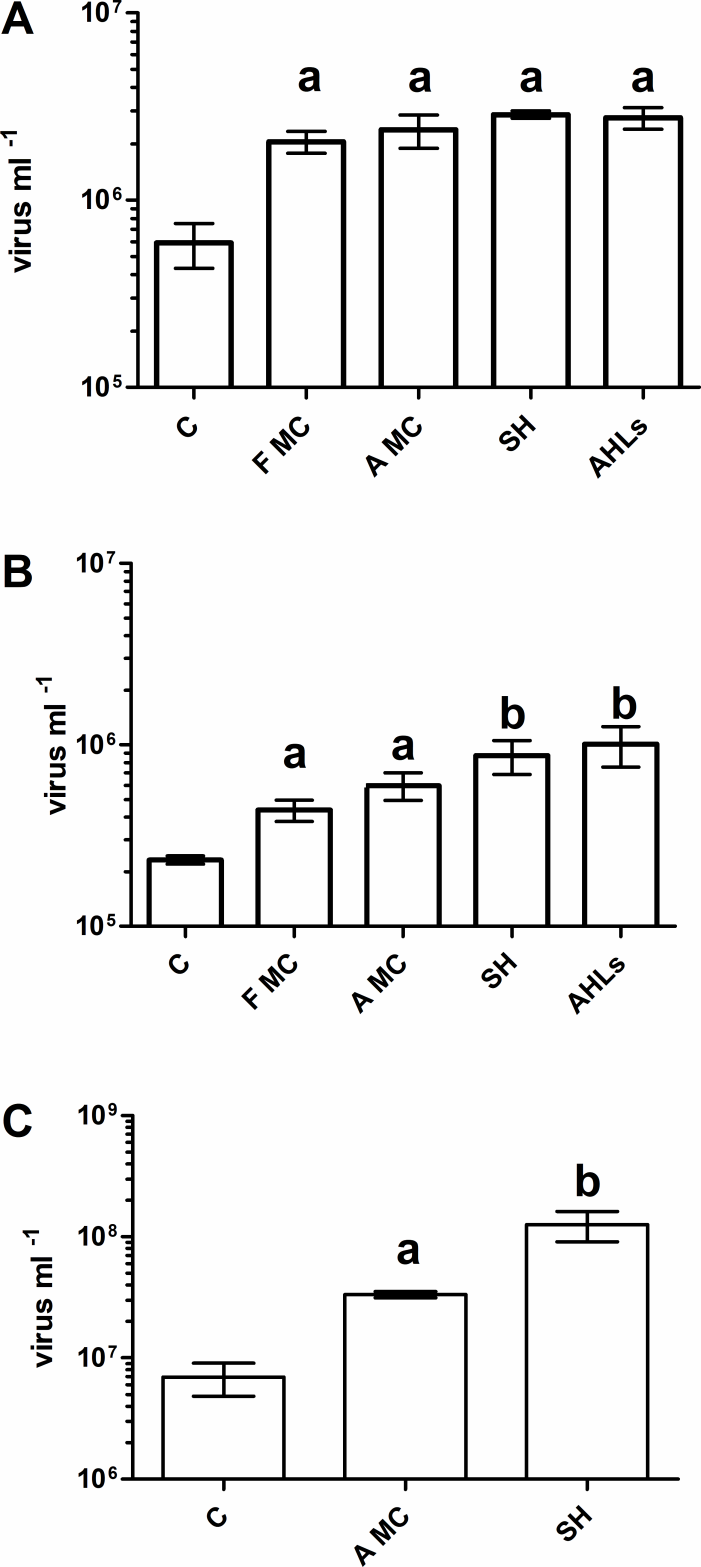
Comparison of inducing agents. (A) Extracted soil bacteria; (B) freshwater bacteria; (C) *E. coli* (λ); error bars represent standard deviation (*N* = 3); lower-case letters indicate statistically significant groups (*P* < 0.05). Treatment groups: C, control; F MC, Fisher brand mitomycin C; A MC, ApexBio brand mitomycin C; SH, SedgeHammer.

For bacteria extracted from soil, all inducing agents generated viral direct counts significantly higher than controls (Fig. 1A). No significant differences were observed across the inducing agents tested. For freshwater bacteria, all inducing agents generated viral direct counts significantly higher than controls (Fig. 1B). SedgeHammer and AHLs induced significantly more phage particles than did the Fisher brand mitomycin C or the ApexBio brand mitomycin C. There was no significant difference between the number of prophages induced based on the source of mitomycin C, nor was there a difference in the number of prophages induced between SedgeHammer and AHLs. For *E. coli* (λ) lysogens, both ApexBio mitomycin C and SedgeHammer generated significantly higher viral direct counts than controls (Fig. 1C). SedgeHammer induced significantly more prophage than did ApexBio mitomycin C.

### Seasonal variation in soil microbial abundance and inducible lysogeny

**Fig 2:**
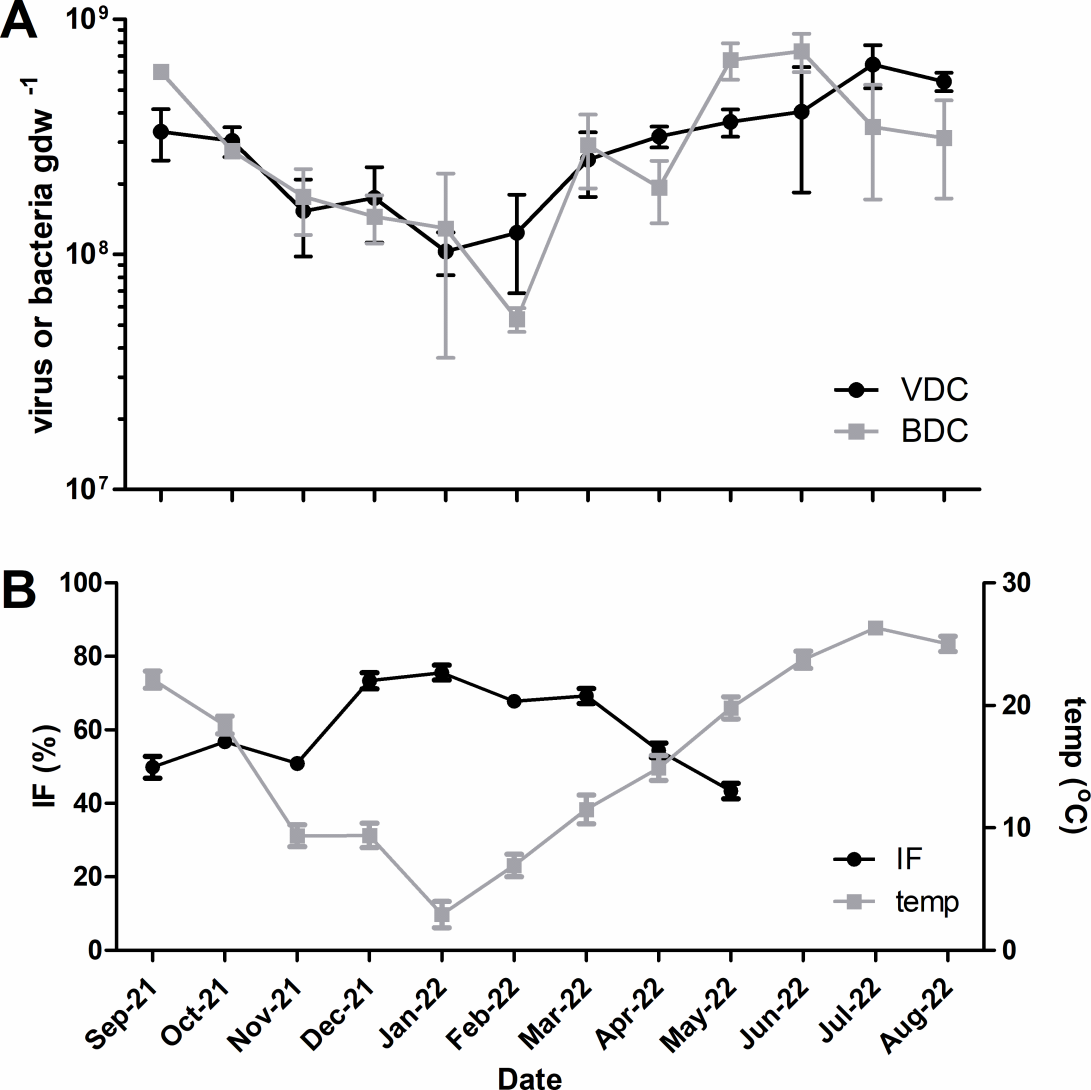
Soil microbial dynamics over the annual observation period. (A) Soil viral and bacterial abundance, per gram dry weight of soil. Black circles represent average monthly soil viral abundance; gray squares represent average monthly soil bacterial abundance; error bars represent standard deviation (*N* = 3); (B) Inducible fraction based on calculated burst size (black circles) and average monthly air temperature (gray squares); error bars represent standard deviation (*N* = 3 for IF, *N* = 28–31 for temperature).

A distinct seasonal trend was observed in soil viral and bacterial abundance over time (Fig. 2A). Both bacterial and viral abundance were higher in the warmer months and lower in the colder months; indeed, a significant positive correlation was observed between soil bacterial abundance and average monthly air temperature (*r* = 0.853, *P* = 0.0004; Table 3) and between soil viral abundance and average monthly air temperature (*r* = 0.986, *P* > 0.0001; Table 3). No significant relationships were observed between microbial abundances and soil physical/chemical properties (Table 3).

**TABLE 3:** Spearman correlations^*a*^

	*P*_*i*_	*B*_*l*_	*B*_*z*_	VA	BA	IF	Rain	Temp	%W	OM	K	Mg	Ca	pH	CEC	NO_3_
Pi		**0.017**	**0.005**	**0.002**	**0.028**	**0.001**	0.983	**0.004**	0.075	0.321	0.446	0.758	0.681	0.922	0.513	0.568
Bl	**0.671**		0.351	**0.011**	0.101	**>0.001**	0.812	**0.020**	0.090	0.786	0.532	0.186	0.331	0.827	0.665	0.910
Bz	**0.746**	0.296		0.053	0.232	0.125	0.569	**0.049**	0.375	0.306	0.500	0.242	**0.046**	0.634	0.187	0.263
VA	**0.797**	**0.699**	0.570		**>0.001**	**>0.001**	0.308	**>0.001**	0.138	0.381	0.868	0.732	0.557	0.991	0.308	0.799
BA	**0.629**	0.497	0.373	0.853		**0.002**	0.226	**>0.001**	0.131	0.272	0.895	0.497	0.471	0.991	0.175	0.608
IF	**0.831**	**0.852**	0.468	**0.887**	**0.789**		0.845	**>0.001**	0.016	0.443	0.806	0.842	0.965	0.869	0.510	0.909
rain	0.007	0.077	0.183	0.322	0.378	0.063		0.265	0.131	0.293	0.188	0.158	0.430	0.090	0.031	0.240
temp	**0.762**	**0.657**	**0.577**	**0.986**	**0.853**	**0.873**	0.350		0.124	0.474	0.649	0.618	0.484	0.844	0.308	0.955
%W	0.531	0.510	0.282	0.455	0.462	0.676	0.462	0.469		0.862	0.982	0.956	0.897	0.150	0.665	0.403
OM	0.313	0.088	0.323	0.278	0.345	0.245	0.331	0.229	0.056		0.916	0.152	0.135	0.186	0.005	0.099
K	0.243	0.200	0.216	0.054	0.043	0.079	0.408	0.147	0.007	0.034		**0.011**	0.053	0.754	0.290	0.794
Mg	0.100	0.410	0.366	0.111	0.218	0.065	0.435	0.160	0.018	0.440	0.704		**>0.001**	0.951	0.003	0.642
Ca	0.133	0.308	**0.585**	0.189	0.231	0.014	0.252	0.224	0.042	0.458	0.569	0.899		0.590	0.013	0.843
pH	0.032	0.071	0.153	0.004	0.004	0.053	0.510	0.064	0.443	0.410	0.101	0.020	0.173		0.088	0.093
CEC	0.210	0.140	0.408	0.322	0.420	0.211	0.622	0.322	0.140	0.746	0.333	0.777	0.692	0.513		0.253
NO_3_	0.184	0.037	0.351	0.083	0.165	0.037	0.367	0.018	0.266	0.499	0.085	0.150	0.064	0.506	0.358	

^*a*^ Top triangular matrix, *P*-values; bottom triangular matrix, Spearman’s rho (*r*); significant values are in bold. *P_i_*, prophage induced; *B_l_*, bacteria lysed; *B*_*z*_, burst size; VA, ambient soil viral abundance; BA, ambient soil bacterial abundance; IF, inducible fraction based on calculated burst size; rain, mean monthly rainfall; Temp, mean monthly air temperature; %W, soil gravimetric water content; OM, soil organic matter content; and CEC, soil cation exchange capacity.

Significant differences in viral direct counts were observed between mitomycin C (ApexBio) treated samples and untreated controls for the samples collected in September 2021 through May 2022, but not for June, July, or August 2022 (Table 2). As a result, the inducible fraction of soil bacteria based on calculated burst size (IF_BZ_) varied from undetectable (i.e., lack of significant prophage induction in mitomycin C-treated samples) to 75.57%, with the largest proportion of lysogens observed in winter (December and January, Table 2; Fig. 2B). The inducible fraction using an assumed burst size of 20 [IF_20_ (26)] exhibited the same general trend of seasonality in inducible lysogeny as compared to IF_BZ_, but with lower proportions of lysogens comprising the bacterial community (1.81%–15.93%), and a relatively steady proportion of inducible lysogens (14%–15%) observed from fall through early spring (October through March, Table 2; Fig. 2B). Both IF_BZ_ and IF_20_ indicate a distinct seasonal trend in inducible lysogeny for the bacterial community within the soil of study (Table 2; Fig. 2B). A strong negative correlation was observed between IF_BZ_ and the average monthly air temperature (*r* = −0.873, *P* = 0.0002; Table 3). In addition to temperature, IF_BZ_ was negatively correlated with ambient soil viral abundance (*r* = −0.887, *P* = 0.0001) and bacterial abundance (*r* = −0.788, *P* = 0.0023) but was not correlated with any of the measured soil physical/chemical properties (Table 3). A strong positive correlation was observed between the number of prophages induced and bacteria lysed (*r* = 0.671, *P* = 0.0168; Table 3).

The calculated burst size (*B*_*Z*_) varied from 0.73 to 8.33 (Table 2) and a weak negative correlation was found between *B*_*Z*_ and average monthly air temperature (*r* = −0.577, *P* = 0.049; Table 3). Not surprisingly, a significant positive correlation was observed between the number of prophages induced and *B*_*Z*_ (*r* = 0.746, *P* = 0.005; Table 3). *B*_*Z*_ was significantly correlated with soil calcium (*r* = 0.584, *P* = 0.0459; Table 3), but not with any other soil physical/chemical properties (Table 3). Significant negative correlations were found between the number of prophages induced and ambient soil viral abundance (*r* = −0.797, *P* = 0.0019) and between the number of prophages induced and ambient soil bacterial abundance (*r* = −0.629, *P* = 0.0283; Table 3).

## DISCUSSION

The main goal of this work was to elucidate potential seasonal trends in lysogeny in soil bacterial communities. Because of inconsistencies in earlier attempts at monthly induction assays within this study, we investigated whether the source and type of inducing agent impacted the efficacy of prophage induction. Once we confirmed our choice of inducing agent and implemented key steps in the assay to reduce both background viral abundance and continued cell growth (detailed below), we were able to detect a definitive seasonal trend in lysogeny within the soil bacterial community.

### Technical considerations

A recent meta-analysis of predominantly aquatic induction studies has raised questions about the overall utility of chemical induction assays in drawing meaningful conclusions about lysogeny within environmental bacterial populations (14). With these questions in mind, the present work sought to reduce sources of error and account for variation as much as reasonably possible. In carrying out chemical induction assays, we found it critical to include steps that reduced background virus particles in bacterial preparations used in induction assays. Particularly, in working with the *E. coli* (λ) system, autoinduction was a persistent problem. As others have noted, this could be resolved by pelleting bacterial cells and resuspending them in a fresh medium (18, 41). The removal of pre-existing virus particles from the medium prior to induction greatly enhanced the ability to detect prophage induction (i.e., an increase in extracellular virus particles in mitomycin C-treated samples). We further sought to reduce spurious induction by limiting the exposure of samples to light of any kind. In all induction experiments, all replicates, both treatments and controls, were incubated in the dark by covering tubes with aluminum foil. While we did not perform any experiments to quantitatively compare these dark-incubated samples with the ones that were exposed to ambient (fluorescent) lab lighting, we felt this step was worth noting.

An important problem in interpreting the outcomes of chemical induction assays is that changes in bacterial direct counts are assumed to be solely due to induction-mediated cell lysis. This assumption ignores bacterial mortality due to other factors (e.g., metabolic poisoning from the inducing agent and the release of viral particles from lytic infections) that potentially decrease estimates of burst sizes and of the lysogenic fraction. It is also possible for bacterial cells to continue dividing even as prophage induction progresses, paradoxically leading to increases in bacterial direct counts concomitant with increases in virus counts. This possibility poses eminent challenges to interpreting inducible fraction in various bacterial communities based on the present mathematical equations. The resuspension of cells in M9 salts has commonly been used to starve cells, restricting bacterial growth and division (43, 44). In the present study, bacterial cells were resuspended in M9 salts prior to carrying out induction assays. Not only did this reduce the number of background virus particles in all samples but also the replacement of growth medium or soil extract with M9 salts should inhibit further cell growth and slow the production of obligate lytic virus particles. The strong positive correlation observed between the number of prophages induced and bacteria lysed (*r* = 0.6713, *P* = 0.01683; Table 3) supports the idea that increases in viral direct counts in mitomycin C-treated samples were due to prophage induction.

### Comparison of inducing agents

While mitomycin C remains the gold standard for prophage induction assays, a key question of the present study was whether the source of mitomycin C is a determining factor in the outcomes of chemical induction assays. Almost all previous induction studies have sourced their mitomycin C from Fisher Scientific (now ThermoFisher, Waltham, MA, USA). In our initial attempts to perform monthly induction assays using natural soil bacterial extracts, we had sourced mitomycin C from ApexBio (Houston, TX, USA) due to significant cost savings compared to Fisher, but the results from these induction assays were highly inconsistent (data not shown). Thus, as part of our comparison of inducing agents, we addressed the question of whether the source of mitomycin C affected the outcome of prophage induction assays (Fig. 1A and B).

In addition to mitomycin C, there has long been interest in identifying ecologically relevant chemicals that may also trigger prophage induction in natural bacterial populations. Compounds including aliphatic and aromatic hydrocarbons (45), commercial sunscreens (35), and the quorum-sensing molecules, acyl-homoserine lactones (13) have all been experimentally shown to induce prophages from environmental bacterial assemblages. These compounds may represent a more realistic trigger of induction in natural environments, as a main criticism of mitomycin C is that it is unlikely to be found naturally in these environments.

Given that environmental pollutants may act as prophage-inducing agents (33), part of this work examined the inducing potential of SedgeHammer, an herbicide commonly used on the William and Mary campus to treat nutsedge and other weeds. The active ingredient in SedgeHammer is halosulfuron methyl, which inhibits acetolactate synthase, eventually causing a halt in DNA replication. This impairment of normal DNA replication may activate the SOS repair system and subsequent prophage induction. Previous, unpublished work suggested that SedgeHammer acts as an inducing agent for freshwater bacteria (37), but the potential impacts on soil lysogens remain unknown. Thus, another part of our comparison of inducing agents challenged both freshwater and soil bacteria, as well as the *E. coli* (λ) lysogen model, with SedgeHammer to test its inducing potential (Fig. 1A through C). Finally, we sought to test previous reports that Gram-negative quorum-sensing molecules, AHLs, can induce prophages from environmental bacterial assemblages (Fig. 1A and B).

The results of the experiments comparing inducing agents led to several important conclusions. (i) We found no difference in induction based on the source of mitomycin C (Fig. 1A and B). (ii) ApexBio mitomycin C generated strong and significant induction responses in all bacterial populations tested (Fig. 1A through C). (iii) The herbicide SedgeHammer (halosulfuron methyl) is a potent prophage-inducing agent (Fig. 1A through C). (iv) AHLs appear to be strong inducing agents for natural populations of both freshwater and soil bacteria (Fig. 1A and B). Given that the induction responses generated by ApexBio mitomycin C were not significantly different from the responses generated by Fisher mitomycin C (Fig. 1A and B), all further induction assays in this study were carried out using ApexBio mitomycin C.

### Seasonal variation in soil microbial abundance

In the present study, a clear seasonal trend was observed between soil viral and bacterial abundance and average monthly air temperature (Table 3), with the lowest abundances in winter and the highest abundances in summer (Fig. 2A). In addition, soil viral abundance and bacterial abundance were not only strongly and significantly correlated (Table 3), but viral abundance was rarely higher than bacterial abundance at any given sampling time (Fig. 2A). This stands in sharp contrast to seasonal viral abundance trends reported in other studies.

One study compared microbial abundances over an annual cycle in three soils in Oklahoma, USA (46). Viral abundances were generally in the 10^8^–10^9^ gdw^−1^ range over the annual cycle, while bacterial abundances ranged from 10^5^ to 10^9^ gdw^−1^, with viral abundances typically exceeding bacterial abundance by at least 10-fold. Viral abundance was strongly correlated with leaf area index (*r* = 1.0) for crop soils and with soil ammonium for one of the soils (*r* = −0.56), but not with any other measured factors (46).

Another study compared viral abundances over an annual cycle in four soils in Thuringia, Germany (47). When temporal correlations were examined between viral abundance and edaphic factors in each individual soil, no single factor was universally correlated across all four soils in the study. For one of the two forest soils, a significant positive correlation was observed between viral abundance and air temperature (*r* ~ 0.47), while no correlation with temperature was found for the other forest soil in this study. Meanwhile, both the pasture and cropland soils exhibited negative correlations with temperature (*r* = −0.39 and *r* = −0.57, respectively) (47).

In the only other available study on temporal dynamics of viral abundance in soils, researchers compared viral and bacterial abundance at five sites at Kellogg Biological Station in Michigan, USA (27). Viral abundance in cropland soils did not vary by more than about 0.1 log unit over the observation period; for early successional and grassland soils, viral abundance increased in late summer into September, then decreased through November; for the forest soil, viral abundance was mostly steady at ~2 × 10^8^ gdw^−1^ with slight decreases in June and November. For all five soils in this study, viral abundance was consistently higher than bacterial abundance, generally by a factor of 5. The measured factors that correlated strongest with viral abundance across all soils in the study were soil organic carbon (*r* = 0.42) and total nitrogen (*r* = 0.39).

Including data from the present study, the results of these studies on temporal dynamics of viral abundance in soils are highly variable. Trends in viral abundance and relevant associated factors appear to depend heavily upon the individual soil, land use, and management practices (27, 46, 47). In this regard, whether or not viral abundance trends and their correlates may be similar between similar soil types (e.g., two cropland soils under the same management and rotation or two forest soils of the same parent material and vegetation) remains to be seen.

### Seasonal variation in inducible lysogeny

Currently, only one other comparable study that measures the inducible fraction of soil bacteria over time is known (27). This study compared five sites at the KBS in Michigan, USA: two crop fields planted to corn-soybean-wheat, one forested site, one early successional site, and one grassland site. Researchers collected monthly soil samples from May to November and used mitomycin C to induce prophages from extracted soil bacteria. On one hand, prophage induction was observed in all time points from all soils, with viral abundances in mitomycin C-treated samples exceeding those of controls by 180%–400%. On the other hand, determinations of the IF_BZ_ of soil bacteria were difficult because the calculated burst sizes in this study were frequently less than one phage per induced cell, while the use of an assumed burst size of 20 (IF_20_) resulted in lower inducible fractions than previously reported for soils (27). For the forest soil used in the KBS study, prophage induction was highest in early summer (June, exhibited ~600% higher viral abundance than controls) and mid-fall (October, exhibited ~400%). Unfortunately, samples were not collected or analyzed during the winter or early spring months (December–April).

In the present study, prophage induction was weakest in the summer months, dropping from 79% in May to 7% in August (Table 2). While the IF_BZ_ was highest in December–March (Table 2, Fig. 2B), the highest prophage inductions relative to controls were in October–November (~2,000%, Table 2). With the exception of the September sample, none of the calculated burst sizes in the present study were <1 and varied from ~4 to 8 (Table 2). While the calculation of inducible fraction involves certain assumptions (e.g., the increase in virus particle abundance in mitomycin C-treated samples is accompanied by a decrease in bacterial cell counts due to induction) and is susceptible to erroneous calculations of burst size, a clear lack of prophage induction was nevertheless observed during the summer months in the present study (Table 2). This lack of significant induction correlates with the highest air temperatures, the highest soil viral abundance measures, and the highest bacterial abundance measures in this annual cycle (Fig. 2). In this regard, the observed dynamics are similar to those reported for productive coastal waters, in which mitomycin C-inducible lysogeny was greatest in the colder, winter months, and often not detectable in the warmer, summer months (17, 18, 45).

The biological relevance of inducible fraction calculations across published environmental induction studies has been questioned (14). In this critical analysis, the authors found that across the literature, one-third of the total reported IF values were negative. Furthermore, some reported IF values were inconsistent even within technical replicates in the same study. Because of the frequency with which biologically nonsensical values have been reported, the ways in which the inducible fraction of bacteria is calculated should be carefully considered and heavily scrutinized. As mentioned above (see Technical considerations), several steps were taken in the present study to improve the robustness of our results. Chief among these was washing cells with M9 salts to both remove background virus particles and remove nutrients for cell growth during the induction incubation period. It is worth noting the strong agreement and consistency between technical replicates (as evidenced by reported error terms) and reiterating the strong positive correlation observed between the number of prophage induced and bacteria lysed (*r* = 0.6713, *P* = 0.01683; Table 3) supporting the idea that increases in viral direct counts in mitomycin C-treated samples were likely due to prophage induction.

### Conclusions and future directions

There are many ways in which future studies could build off of this work. First, we suggest incorporating viral reduction approaches into sample processing prior to induction. Washing cells in a nutrient- and virus-free medium (e.g., M9 salts) should reduce the number of free virus particles in the sample before induction occurs. This may generate a higher signal-to-noise ratio, in that the difference in viral direct counts between controls and treatments is more obvious. The use of a nutrient-free buffer for cell washing and resuspension also deprives bacteria of nutrients for continued growth, hampering further cell division and clarifying the potential link between changes in viral and bacterial direct counts. Virus reduction approaches may also limit the amount of background noise apparent under epifluorescence microscopy, leading to clearer fields of view and more consistent results. One potential drawback to this approach is that some bacterial cells may be lost in the washing process. Nevertheless, the consistency of results in the present study is a strong argument for its incorporation in future work.

The emerging picture of viral dynamics within soils is murky. While the present study suggests a clear seasonal pattern both for soil viral abundance and inducible lysogeny within an Appalachian oak-hickory forest soil bacterial community, this same pattern has not been observed for other soils. The unknown degree to which our results may be extrapolated to other soils remains a key limitation of this study. While previous work suggests that lysogeny may be quite common in soils (26–28, 48), its importance as a mechanism for phage replication and persistence within soil microbial communities remains poorly understood. Overall, our results implicate the halosulfuron methyl herbicides as a potent and ecologically relevant inducing agent for soil bacteria. Additionally, our findings support previously established paradigms that lysogeny is favored when host metabolism is slower (lower temperatures) and when host cells are less abundant and make an important contribution to the limited knowledge about trends in lysogeny in soil microbial communities across the globe.

## Data Availability

Upon acceptance, raw data regarding viral particle counts and bacterial cell counts for all experiments will be provided via figshare (10.6084/m9.figshare.23971818).

## AFTER EPUB

*[This article was published on 12 December 2023 with a standard copyright line (“© 2023 American Society for Microbiology. All Rights Reserved.”). The authors elected to pay for open access for the article after publication, necessitating replacement of the original copyright line with a CC BY 4.0 copyright line, and this change was made on 22 December 2023.]*
